# Oncostatin M and TLR-4 Ligand Synergize to Induce MCP-1, IL-6, and VEGF in Human Aortic Adventitial Fibroblasts and Smooth Muscle Cells

**DOI:** 10.1155/2013/317503

**Published:** 2013-11-06

**Authors:** David Schnittker, Karen Kwofie, Ali Ashkar, Bernardo Trigatti, Carl D. Richards

**Affiliations:** ^1^McMaster Immunology Research Centre, Department of Pathology and Molecular Medicine, McMaster University, ON, Canada L8S 4K1; ^2^Department of Biochemistry and Biomedical Sciences, Thrombosis and Atherosclerosis Research Institute, David Braley Cardiac, Vascular and Stroke Research Institute, McMaster University, Canada

## Abstract

Accumulating evidence suggests that adventitial fibroblasts play a significant role in contributing to inflammation of the arterial wall and pathogenesis of atherosclerosis. The effects of gp130 cytokines on these cells (including oncostatin M-[OSM] and IL-6), some of which have been implicated in atherosclerosis, are currently unknown. Experiments were performed to determine whether gp130 cytokines regulate human aortic adventitial fibroblasts (HAoAFs) or smooth muscle cells (HAoSMCs) alone or in context of TLR-4 ligands (also implicated in atherosclerosis). HAoAFs and HAoSMCs were stimulated with LPS and/or one of OSM, IL-6, IL-11, IL-31, or LIF. ELISAs performed on cell supernatants showed that stimulation with OSM alone caused increased MCP-1, IL-6, and VEGF levels. When combined, LPS and OSM synergized to increase MCP-1, IL-6, VEGF protein, and mRNA expression as assessed by qRT-PCR, in both HAoAFs and HAoSMCs, while LPS-induced IL-8 levels were reduced. Such effects were not observed with other gp130 cytokines. Signalling pathways including STATs, MAPKinases, and NF**κ**B were activated, and LPS induced steady state mRNA levels of the OSM receptor chains OSMR**β** and gp130. The results suggest that OSM is able to synergize with TLR-4 ligands to induce proinflammatory responses by HAoAFs and HAoSMCs, supporting the notion that OSM regulation of these cells contributes to the pathogenesis of atherosclerosis.

## 1. Introduction

 Atherosclerosis is a main cause of cardiovascular disease (CVD) which is a leading factor in determining both morbidity and mortality in developed countries. Atherosclerosis is characterized as an inflammatory process which occurs as a result of complex cellular and environmental interactions [1]. Atherosclerotic lesion development involves the expression of cytokines and growth factors by infiltrating inflammatory cells such as macrophages and by structural local cells of the vessel wall. A strong emphasis has been placed on the roles of endothelial cells (ECs) and smooth muscle cells (SMCs) of the intima and media, respectively [1]; however, there is growing evidence to suggest that cells of the adventitia may also play a role in the generation of atherosclerotic plaques. Adventitial fibroblasts have been shown to be involved in neointima formation following vascular insult [2, 3] and the generation of reactive oxygen species [4] and have the capacity to attract leukocytes and release cytokines, chemokines, and growth factors [5, 6]. Inflammatory molecules such as MCP-1 [7], IL-6 [8], VEGF [9], and IL-8 [10] have been implicated in atherosclerosis; however, their regulation and expression by adventitial fibroblasts remain to be fully defined.

 Among the network of molecules known to modulate inflammation in general are several within the gp130 family of cytokines that include interleukin (IL)-6, IL-11, and IL-31, oncostatin M (OSM), leukemia inhibitory factor (LIF), ciliary neurotrophic factor (CNTF), IL-27, and others [11]. There is some evidence that suggests that gp130 cytokine members OSM, and IL-6, may play a role in atherosclerosis. OSM and IL-6 have been detected within ApoE deficient mouse and human atherosclerotic plaques [12–14]. Furthermore, stimulation of human endothelial cells *in vitro *with either OSM or IL-6 resulted in increased expression of leukocyte adhesion molecules and chemokines [15, 16], which have the potential to promote leukocyte infiltration. In addition, OSM can amplify the effects of other inflammatory mediators in other systems; OSM has been shown to synergize with various cytokines such as TNF*α*, IL-1*α*, and with IL-17 to induce cartilage breakdown [17–19]. In human airway smooth muscle cells, the synergistic actions of OSM with IL-1*β* resulted in increased chemokine, growth factor, and cytokine expression [20]. The effects of OSM on aortic smooth muscle cells are only recently emerging, and effects on aortic adventitial fibroblasts are not known. 

 Toll-like receptors (TLR) are established as important sensing molecules of the innate immune system, and cells that express TLRs can respond with inflammatory signalling pathways. TLR-4 ligands, receptor complexes, and cell signalling pathways have been well characterized. Interestingly, TLR-4 has been detected on numerous cell types within atherosclerotic lesions, and its agonists have also been detected in atheromas (reviewed by den Dekker et al. [21]). In addition, double knockout ApoE −/− TLR-4 −/− mice demonstrated a significant reduction in atherosclerosis compared to ApoE −/− mice [22], and the administration of LPS (a typical TLR-4 ligand) to the adventitial surface of murine arteries increased atherosclerosis compared to control, implicating the adventitia in lesion progression [23]. However, precise mechanisms are not yet clear, and how TLR systems and gp130 cytokines interact is not known. It is therefore of interest to elucidate the effects of OSM on mesenchymal cells of the aorta, including adventitial fibroblasts and smooth muscle cells, in context of gp130 cytokines or other stimuli, in order to delineate potential roles of regulation of structural cell activation in plaque development. We here used *in vitro* cell culture systems of human aortic cells and showed induction of synergistic responses, cell signalling, and receptor regulation by OSM and TLR-4 ligand in aortic adventitial fibroblasts and smooth muscle cells.

## 2. Materials and Methods

### 2.1. Cell Culture

Human aortic adventitial fibroblasts (HAoAFs) and human aortic smooth muscle cells (HAoSMCs) were primary cells purchased from Lonza Group Ltd. (Basel, Switzerland) and were cultured in stromal cell growth medium or smooth muscle growth medium-2 (Lonza Group Ltd.), respectively, according to the manufacturer's instructions in 5% FBS, at 37°C in 5% CO_2_ conditions. Cells were stimulated *in vitro* upon reaching approximately eighty percent confluency. Cultures were serum deprived in media containing 2% FBS for three hours prior to stimulation and then stimulated with media containing 2% FBS for designated time points with recombinant OSM, LIF, IL-6, IL-31, or IL-11 (R&D Systems, Minneapolis, MN) and/or with lipopolysaccharide (Sigma Aldrich Corp., St. Louis, MO). Experiments were performed on cells between passages 3 and 8. LPS from *Escherichia coli* 026:B6 (highly purified, free of other TLR ligands) was purchased from Sigma-Aldrich Canada (Oakville, ON).

### 2.2. Enzyme Linked Immunosorbent Assays and Immunoblots

ELISAs were performed for IL-6, VEGF, and IL-8 using DuoSet kits purchased from R&D Systems. MCP-1 ELISAs were purchased from Biolegend (San Diego, CA). ELISAs were performed according to the manufacturer's instructions. For analysis of cell signalling pathways, HAoAF and HAoSMC cultures were stimulated for 20 minutes, and cell lysates were generated, and immunoblots were performed as published previously [24]. Primary antibodies for pSTAT-1 (Tyr 701), total STAT-1, pSTAT-3 (Tyr 705 and Ser 727), total STAT-3, pSTAT-5 (Tyr 694), p-p38 (Thr 180/Tyr 182), total p38, p-SAPK/JNK (Thr 183/Tyr 185), p-NF*κ*B p65 (Ser 536), total NF*κ*B p65, pAkt (Thr 308), and total Akt were purchased from Cell Signaling Technology (Danvers, MA). Antibodies for p-ERK 1/2 (E4), total ERK 1/2 (K23), total JNK (D-2), total STAT-5 (C-17), and total Actin (I-19) were purchased from Santa Cruz Biotechnology Inc. Mouse anti-goat IgG-HRP goat anti-rabbit IgG-HRP, or goat anti-mouse IgG-HRP were used as secondary antibodies and were purchased from Santa Cruz Biotechnology Inc. Relative protein levels were determined by densitometry using ImageJ software (NIH, Bethesda, MD). All Western blot densitometry analyses were normalized to a loading control and compared relatively to untreated/control samples.

### 2.3. RNA Purification and Analysis by Real-Time Quantitative PCR (TaqMan)

Pure Link RNA mini kit (Life Technologies, Carlsbad, CA) was used according to the manufacturer's instructions to isolate and purify the RNA. RNA was reversed transcribed using SuperScript II (Life Technologies), and 25 ng of the resultant cDNA was used to measure expression of IL-6, IL-8, VEGF, MCP-1, TLR-4, CD14, OSMR-*β*, gp130, and *β*-Actin using TaqMan predeveloped assay reagents purchased from Ambion (Life Technologies). mRNA expression levels for each sample were performed in triplicate and normalized to *β*-Actin levels in that sample and expressed relative to control (media alone) values. TaqMan was performed using the 7900HT Fast Real-Time PCR System (Life Technologies).

### 2.4. Statistical Analysis

All statistical tests were performed using GraphPad Prism 5 software (GraphPad Software Inc., San Diego, CA). A *P* value less than 0.05 was considered statistically significant. For the ELISA data, two-way analysis of variance (ANOVA) was used to determine significance between LPS alone and LPS combined with one of the gp130 cytokines. For the mRNA data, one-way ANOVA was used to determine significance. In all analyses, Bonferroni's post-test was applied.

## 3. Results

### 3.1. Synergistic Induction of MCP-1, IL-6, and VEGF by HAoAFs in Response to LPS and OSM

**Figure 1:**
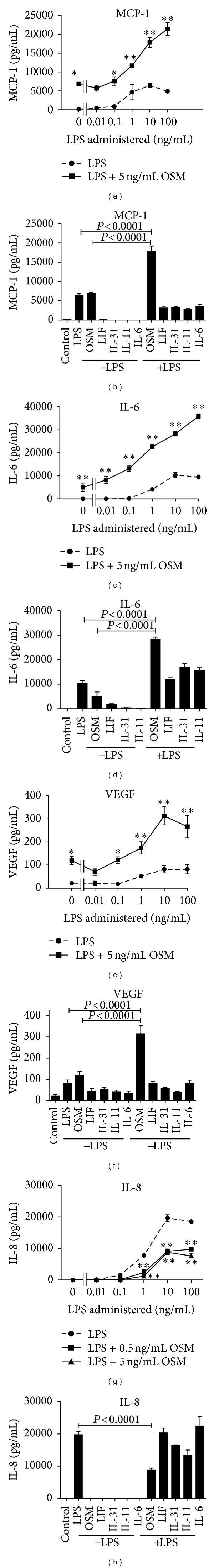
OSM and LPS regulation of MCP-1, IL-6, VEGF, and IL-8 expression by HAoAFs. HAoAFs were cultured as in Section 2 and stimulated with increasing doses of LPS alone or with 0.5 or 5 ng/mL OSM as indicated ((a), (c), (e), and (g)). In ((b), (d), (f), and (h)), cells were stimulated with 5 ng/mL of gp130 cytokine (OSM, LIF, IL-31, IL-11, and IL-6) without or in combination with 10 ng/mL LPS as indicated. Supernatants were collected after 18 hours, frozen (−20°C), and later analyzed by ELISA for MCP-1 ((a), (b)), IL-6 ((c), (d)), VEGF ((e), (f)), and IL-8 ((g), (h)). Results are representative of multiple separate experiments. Statistical significance: *P ≤ 0.05, **P ≤ 0.01, compared to LPS alone at the same concentration.

**Figure 2:**
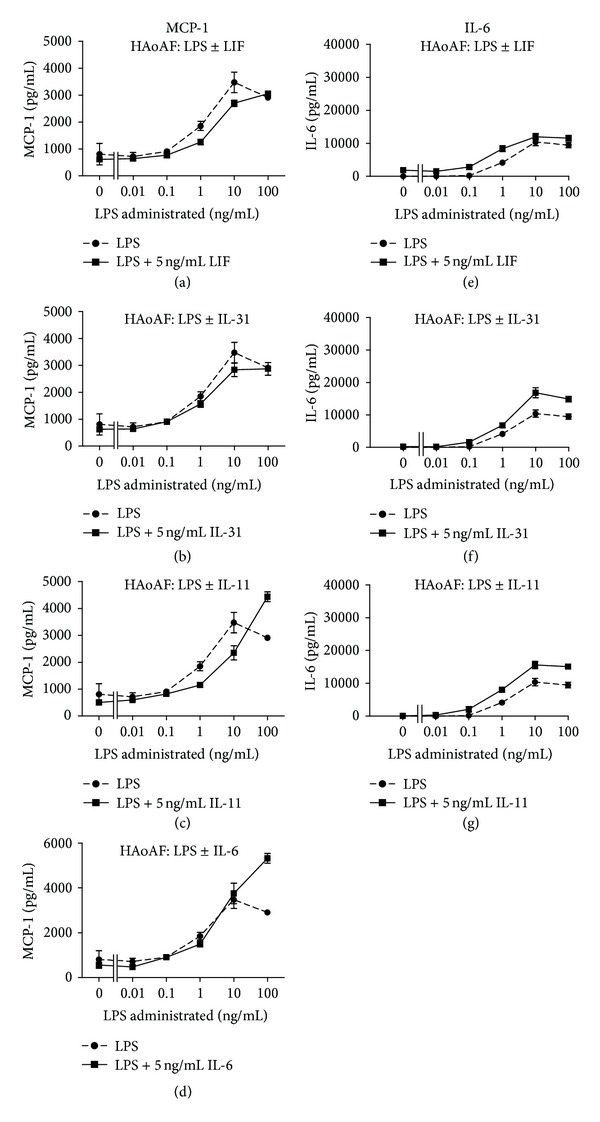
MCP-1 and IL-6 responses to gp130 cytokines by HAoAF. HAoAFs were stimulated with increasing doses of LPS alone or with 5 ng/mL LIF ((a), (e)), IL-31 ((b), (f)), IL-11 ((c), (g)), or IL-6 (d) as indicated. Supernatants were collected and analyzed by ELISA for MCP-1 ((a)–(d)) or IL-6 ((e)–(g)). Results are representative of multiple separate experiments.

Human aortic adventitial fibroblasts were stimulated with the TLR-4 ligand LPS alone, gp130 cytokines alone, or combinations as indicated (Figure 1) to determine responses *in vitro*. MCP-1 levels were measured in the supernatants by ELISA, and significant increases (to over 5000 pg/mL) were detected upon stimulation with LPS or OSM alone (*P* < 0.0001), but not with LIF, IL-31, or IL-6 compared to control levels (Figures 1(a) and 1(b)). Upon stimulation with the combination of LPS and OSM, there was a synergistic induction of MCP-1 (to over 15 000 pg/mL) with the concentrations of 10 and 100 ng/mL LPS (Figure 1(a)), which was not observed with the other gp130 cytokines tested (Figures 1(b) and 2). In contrast, LIF, IL-31, IL-11, and IL-6 showed a trend to suppress the LPS-induced MCP-1 (Figure 1), but this was consistently observed with LIF and IL-11 (and not IL-31 or IL-6) and only at 10 ng/mL (but not other concentrations) of LPS (Figure 2). Collectively, the observations emphasize the stimulatory effect of OSM and not other gp130 cytokines on MCP-1 expression.

**Figure 3:**
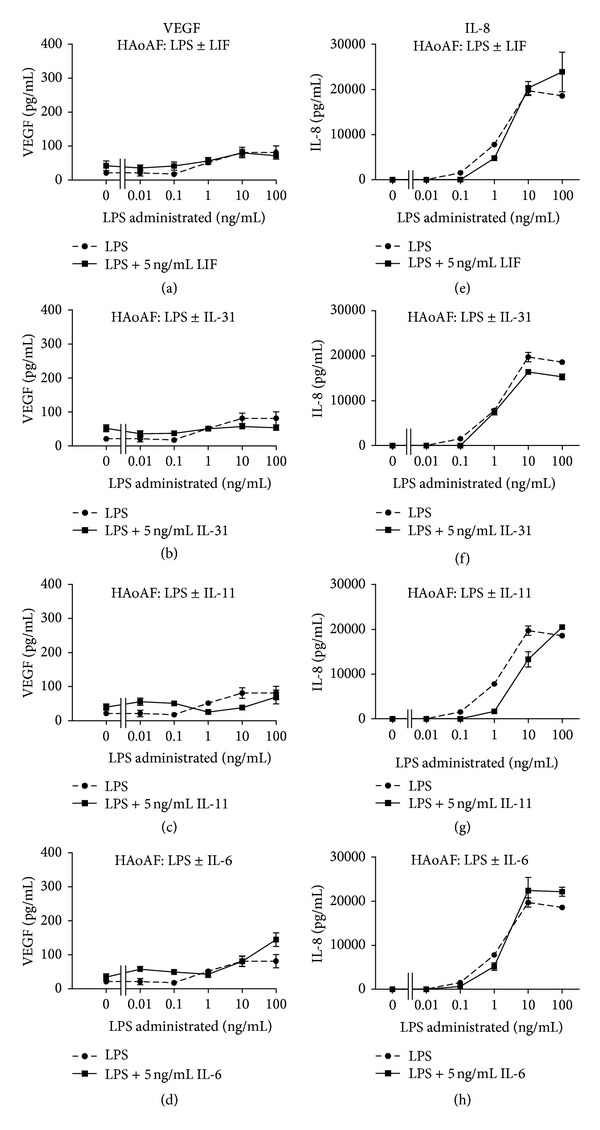
VEGF and IL-8 responses to gp130 cytokines by HAoAF. HAoAFs were stimulated with increasing doses of LPS alone or with 5 ng/mL LIF ((a), (e)), IL-31 ((b), (f)), IL-11 ((c), (g)), or IL-6 ((d), (h)) as indicated. Supernatants were collected and analyzed by ELISA for VEGF ((a)–(d)) or IL-8 ((e)–(g)). Results are representative of multiple separate experiments.

The supernatants were also assayed for IL-6 and VEGF levels. HAoAFs responded to OSM or LPS alone with increases in IL-6 levels detected (Figures 1(c) and 1(d)) in the supernatants after 18 hours (*P* < 0.01 versus control). Furthermore, OSM synergized with LPS in terms of IL-6 induction when the two were used in combination to stimulate HAoAF cells, which was evident at LPS concentrations as low as 0.1 and 1 ng/mL. IL-6 levels were lower in response to other gp130 cytokines tested (Figures 1(d) and 2). In a similar trend, stimulation of HAoAFs with OSM or LPS alone was able to induce significantly increased (*P* < 0.0001) VEGF levels in the supernatants compared to control (Figures 1(e) and 1(f)), and when LPS and OSM were combined, VEGF levels in HAoAF supernatants were enhanced compared to either treatment alone. Combinations of LPS with LIF, IL-31, IL-11, or IL-6 did not have similar effects (Figures 1(f) and 3).

IL-8 levels were also measured by ELISA in HAoAF supernatants and were significantly elevated in response to 1, 10, or 100 ng/mL LPS compared to control (*P* < 0.0001) (Figure 1(g)). Stimulation with OSM alone did not cause an increase in detected IL-8 above control levels (Figure 1(h)). Moreover, when combined with LPS, OSM was able to significantly decrease the IL-8 levels that were detected in HAoAFs supernatants compared to LPS alone (*P* < 0.0001) (Figure 1(g)). Effects of other gp130 cytokine that were tested were not statistically significant (Figures 1(h) and 3). 

### 3.2. Cell Signalling Pathways Activated by LPS, OSM, and OSM/LPS Combination

**Figure 4:**
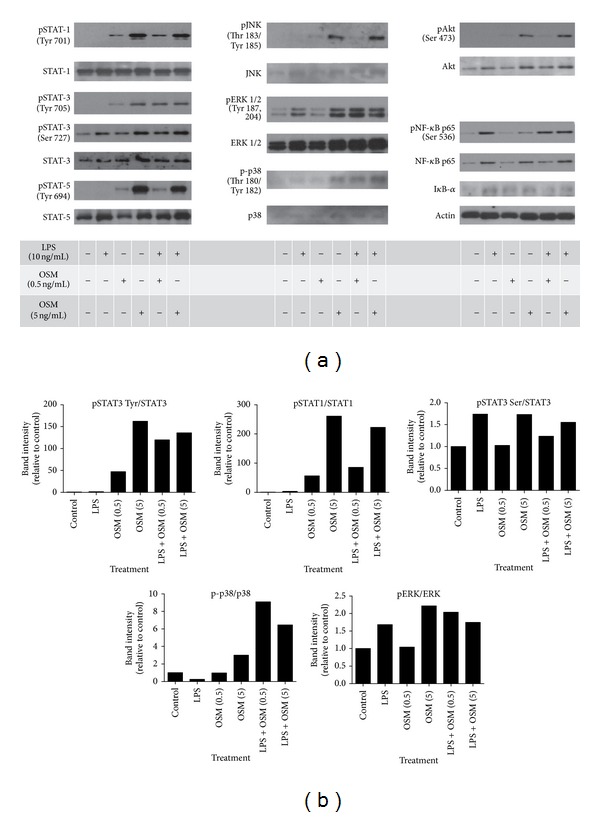
Cell signalling pathways activated in HAoAFs after 20-minute stimulation. HAoAFs were stimulated for 20 minutes with media (control), LPS (10 ng/mL), OSM (either 0.5 or 5 ng/mL), or a combination of LPS (10 ng/mL) and OSM (either 0.5 or 5 ng/mL), and total cell lysates were prepared. Immunoblots were probed with the antibodies indicated (a) and densitometry was used to quantitatively assess signal strength, corrected to total protein for each probe, and expressed normalized to control (unstimulated) levels.

Western blots were generated in order to determine whether there was amplification of intracellular signaling cascades with the combined LPS and OSM stimulation compared to either stimulation alone (Figure 4(a) shows the images, and Figure 4(b) shows the densitometry analysis). At 20 minutes of stimulation, p-tyr-STAT-1, p-tyr-STAT-3 and p-ser-STAT-3, p-tyr-STAT-5, pJNK, pERK, p-p38, and p-Akt showed increased signal compared to control upon stimulation with OSM. LPS stimulation alone induced detectable increases in p-ser-STAT3 and pERK. When the two stimuli were combined, 20 minute signals included similar alterations in of all of these pathways, and a further elevation of p-p38 was evident over that in response to either OSM or LPS alone.

### 3.3. Synergistic MCP-1, IL-6, and VEGF Induction by HAoSMCs in Response to LPS and OSM

**Figure 5:**
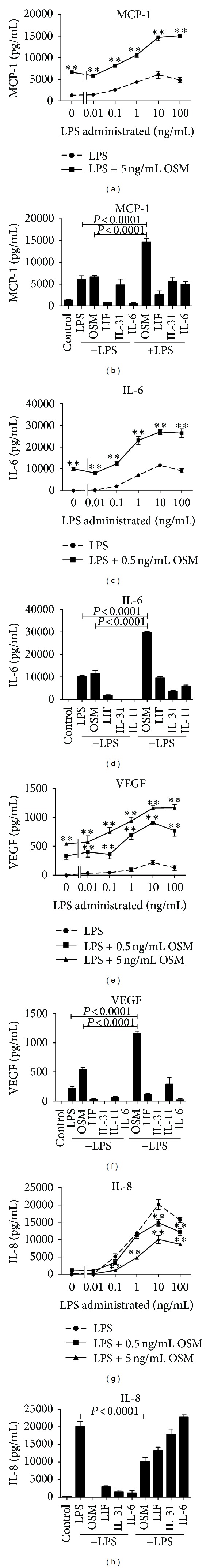
OSM and LPS regulation of MCP-1, IL-6, VEGF, and IL-8 expression in HAoSMC. HAoSMCs were cultured as in Section 2 and then stimulated with increasing doses of LPS alone or with 0.5 or 5 ng/mL OSM as indicated ((a), (c), (e), and (g)). In ((b), (d), (f), and (h)), HAoSMCs were stimulated with 5 ng/mL gp130 cytokine (OSM, LIF, IL-31, IL-11, and IL-6), alone or in combination with 10 ng/mL LPS as indicated. Supernatants were collected after 18 hours, frozen, and later analyzed by ELISA for MCP-1 ((a), (b)), IL-6 ((c), (d)), VEGF ((e), (f)), and IL-8 ((g), (h)). Results are representative of multiple separate experiments. Statistical significance: *P ≤ 0.05, **P ≤ 0.01, compared to LPS alone at the same concentration.

We next examined aortic smooth muscle cells to assess whether they respond in a similar fashion to aortic fibroblasts *in vitro. *Human aortic smooth muscle cell cultures (HAoSMCs) were thus stimulated  with LPS and/or gp130 cytokines in a similar fashion to the experiments with HAoAF. The combined treatment with LPS and OSM resulted in synergistically elevated levels of MCP-1 detected in the supernatants compared to either treatment alone (Figure 5(a)). This effect was unique to cells stimulated with OSM, compared to when LPS was combined with other gp130 cytokines (Figure 5(b)). Upon examining IL-6 levels, OSM alone was able to induce the greatest levels of IL-6 compared to the other gp130 cytokines tested (*P* < 0.0001) (Figure 5(d)). OSM synergized with LPS at doses of 1, 10, and 100 ng/mL LPS in IL-6 induction, when the two stimuli were used in combination (Figure 5(c)), resulting in detection of approximately 3-fold elevation over levels induced by either agent alone (Figure 5(d)). While LPS alone had a small but significant effect on VEGF levels in HAoSMC supernatants compared to control levels (*P* < 0.001), OSM alone induced increased levels of VEGF in a dose dependent manner (Figure 5(e)). In addition, there was a synergistic increase in VEGF levels detected upon stimulation with the combination of LPS and (either 0.5 or 5 ng/mL) OSM. LPS alone induces significant levels of IL-8 in HAoSMC supernatants (0.1 ng/mL LPS *P* < 0.01, 1, 10 and 100 ng/mL LPS *P* < 0.0001 versus control) (Figure 5 (g)), while OSM alone had no effect on detectable levels of IL-8 (Figure 5 (h)). OSM was able to inhibit the IL-8 levels that were induced by LPS when the two stimuli were combined (*P* < 0.0001) (Figures 5(g) and 5(h)).

### 3.4. Augmented MCP-1, IL-6, and VEGF mRNA Levels upon Stimulation with LPS and OSM

**Figure 6:**
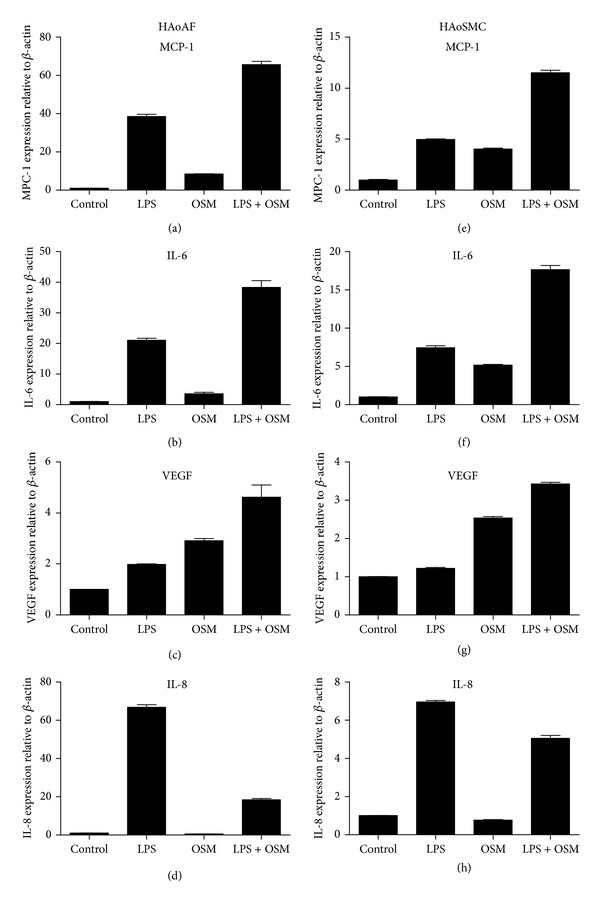
MCP-1, IL-6, VEGF, and IL-8 mRNA levels in HAoAFs and HAoSMCs upon LPS and OSM stimulation. HAoAFs ((a)–(d)) and HAoSMCs ((e)–(h)) were stimulated with LPS (10 ng/mL), OSM (5 ng/mL), or LPS/OSM combination. Steady state levels of MCP-1, IL-6, VEGF, and IL-8 mRNA were measured by qRT-PCR 6 hours after stimulation.

The steady state mRNA levels in HAoAFs and HAoSMCs were also analyzed following stimulation *in vitro* for 6 hours. The combined treatment of LPS and OSM resulted in augmented steady state MCP-1 mRNA levels compared to either treatment alone in HAoAFs and HAoSMCs (Figures 6(a) and 6(e)). As the MCP-1 mRNA levels did, the elevation of IL-6 mRNA also correlated with the data obtained by ELISA. In HAoAFs and HAoSMCs, LPS or OSM alone was able to induce an increase in the steady state IL-6 mRNA levels compared to control, and these levels were further increased upon stimulation with the combination of LPS and OSM (Figures 6(b) and 6(f)). In both cell types, OSM alone was able to induce a greater fold induction of VEGF mRNA than LPS alone, and the combined LPS and OSM treatment resulted in still further elevation of VEGF mRNA steady state levels (Figures 6(c) and 6(g)). As was observed with the IL-8 protein levels, LPS was able to induce an increase in the steady state IL-8 mRNA that was detected compared to control levels in both HAoAFs and HAoSMCs. When LPS was combined with OSM, there was a reduction of the IL-8 mRNA levels detected (Figures 6(d) and 6(h)).

### 3.5. STAT-3 and MCP-1 mRNA Responses to gp130 Cytokines

**Figure 7:**
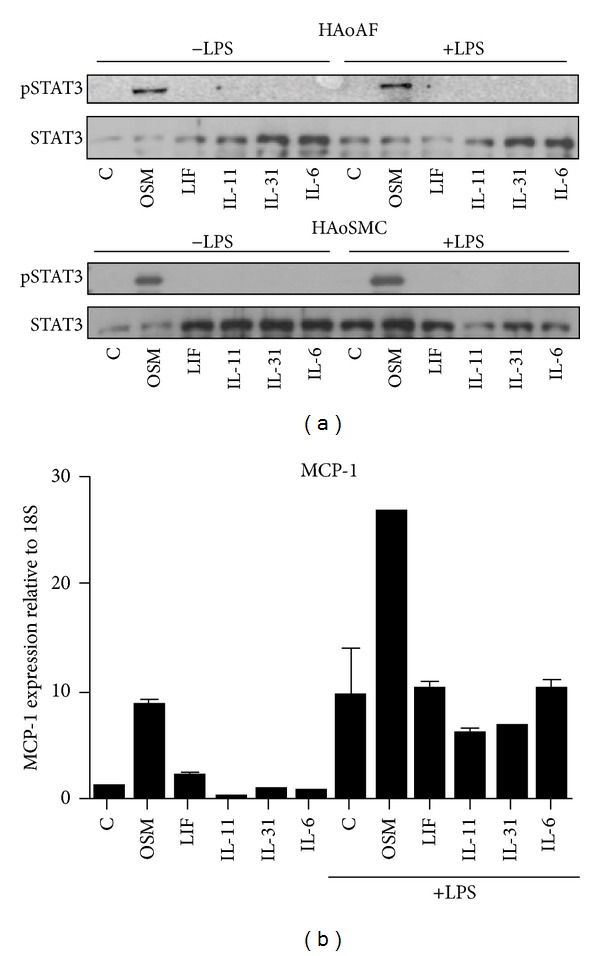
Regulation of STAT-3 and MCP-1 by OSM verses gp130 cytokines. HAoAF (upper panel in (a)) and HAoSMC (lower panel in (a)) were stimulated for 20 minutes with media (control, C) or OSM, LIF, IL-11, IL-31, or IL-6 (all at 5 ng/mL) with or without LPS (10 ng/mL) as indicated. Immunoblots were probed for pSTAT3 and total STAT3 as a loading control. In (b), HAoAFs were stimulated for 6 hours with media alone (control, C) or OSM, LIF, IL-11, IL-31, or IL-6 (all at 5 ng/mL) with or without LPS (10 ng/mL) as indicated. Steady state levels of MCP-1mRNA were measured by qRT-PCR and expressed relatively to housekeeping gene 18S.

To compare the cell signaling of OSM to that of other gp130 cells in HAoAF and HAoSMC, we assessed STAT-3 activation by Western blots as shown in Figure 7(a). Only OSM stimulation resulted in elevated p-STAT3 levels when the cytokines were used at 5 ng/mL concentrations. When HAoAFs were assessed for mRNA levels for MCP-1 at the 6-hour time point (Figure 7(b)), OSM elevated MCP-1 mRNA alone and further in combination with LPS, as previously observed in Figure 6; however, LIF, IL-11, IL-31, or IL-6 did not.

### 3.6. Regulation of OSM Receptor Subunits, TLR4, and CD14 mRNA Levels

**Figure 8:**
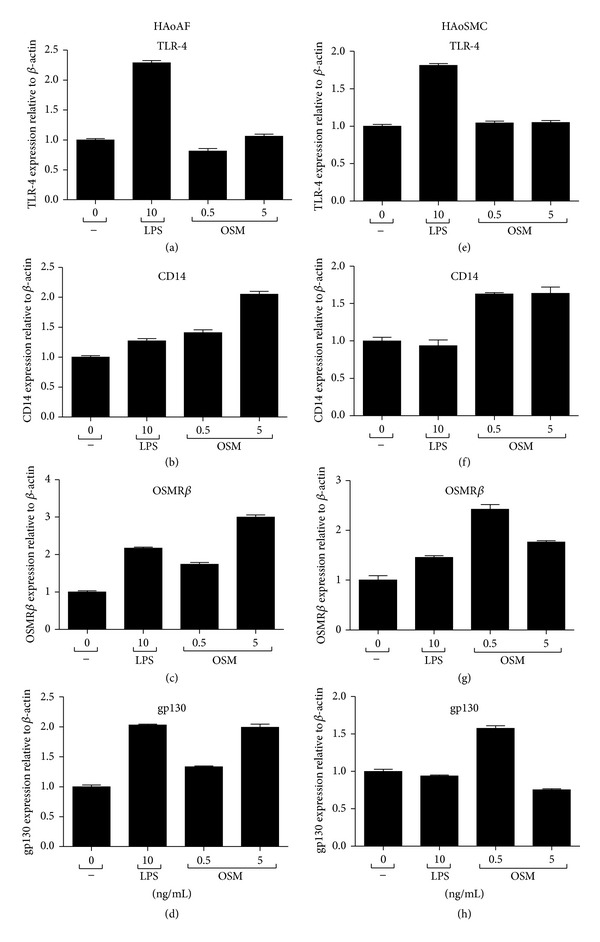
Regulation of steady state mRNA levels of CD14 and OSMR*β*. HAoAFs ((a)–(d)) and HAoSMCs ((e)–(h)) were stimulated with LPS (10 ng/mL) or OSM (0.5, 5 ng/mL), and steady state mRNA levels of TLR-4, CD14, OSMR*β*, and gp130 were measured by qRT-PCR 6 hours after stimulation.

To explore additional mechanisms by which synergistic responses may occur in these cell types in the HAoAFs and HAoSMCs upon stimulation with LPS and OSM, expression of receptors for LPS and OSM was assessed after 5 hours of stimulation. For components of the LPS receptor, TLR-4 and CD14 steady state mRNA levels were measured. LPS stimulation caused an approximately 2-fold induction of TLR-4 expression in both HAoAFs and HAoSMCs, while OSM had no effect compared to control (Figures 8(a) and 8(e)). When CD14 mRNA levels were measured, 5 ng/mL OSM was able to induce a 2-fold increase in steady state levels compared to control in HAoAF cells (Figure 8(b)) and a 1.6-fold increase in HAoSMCs (Figure 8(f)). To determine if LPS modulated the expression of OSM receptor subunits, mRNA levels for the OSMR*β* chain and gp130 chain were assessed. LPS induced approximately 2-fold increases in steady state mRNA levels of both the OSMR*β* and gp130 receptor subunits in HAoAF (Figures 8(c) and 8(d)) where HAoSMCs were less responsive to LPS in this regard (Figures 8(g) and 8(h)). OSM was able to induce increases (greater than 2 fold) in OSMR*β* chain mRNAs in both cell types.

## 4. Discussion

The principal observations made in this study support novel roles of oncostatin M in regulation of inflammatory responses in vessel wall cells and thus atherosclerotic plaques. OSM, but not other gp130 cytokines, induced responses itself and synergized with LPS in regulation of MCP-1, IL-6, and VEGF expression in HAoAF (Figures 1–3). We also observed similar regulation of MCP-1, IL-6 and VEGF in aortic smooth muscle cells (Figure 5), suggesting that simultaneous presence of LPS and OSM engages these cells to potentially contribute to the chemokine and growth factor elevations seen in atherosclerotic plaques. Although SMC activation clearly participates in atherosclerosis [1], in our *in vitro* studies here the levels of MCP-1, IL-6, VEGF, and IL-8 protein detected in the supernatants of HAoAFs were similar or higher on a cell/cell basis than those detected in HAoSMC supernatants. In addition, LPS induced the expression of receptor chains OSMR*β* and gp130, while OSM induced LPS receptor components TLR4 and CD14 in adventitial fibroblasts (Figure 8). The interplay of TLR-ligands, OSM, and OSM receptors (OSMR) represent activities not previously described.

Fibroblast activation occurs in numerous chronic inflammatory diseases, and there is accumulating evidence implicating adventitial fibroblasts in vascular inflammation. Xu et al. showed that adventitial fibroblasts were among the first cells to proliferate and express MCP-1 in aortas of atherosclerotic ApoE knockout mice [25]. Furthermore Tieu et al. [6] demonstrated that IL-6 and MCP-1 were present in the aortic adventitia in close proximity to fibroblasts upon subcutaneous infusion of mice with Angiotensin II, which resulted in macrophage recruitment, adventitial growth, and aortic dissections. Vink et al. have shown that HAoAFs express TLR-4 and can respond to LPS at the mRNA level [23]. The data presented here confirms these results with respect to the expression of MCP-1, IL-6, and IL-8 but further demonstrates expression at the protein level and documents synergy with OSM stimulation. The demonstration that MCP-1 and macrophages were present in atherosclerotic plaques [26] and that MCP-1 causes monocyte/macrophage chemoattraction and extravasation [27] has implicated MCP-1 in the atherosclerotic process. Mice lacking both the LDL receptor and the MCP-1 genes were found to be protected from atherosclerosis compared to LDL receptor knockout mice [7]. Given the functions of MCP-1, the increased expression by adventitial fibroblasts/SMC as a result of a TLR-4 ligand and OSM in the arterial wall could influence atherosclerotic plaque development. 

The role of IL-6 in atherosclerosis is less well defined than that of MCP-1; however, there is evidence that IL-6 can also contribute to vascular inflammation in mouse models [8]. IL-6 has been shown to increase the expression of leukocyte adhesion molecules and chemokines by endothelial cells [16], which could facilitate leukocyte entry into the vessel wall, and thus, synergistic induction of IL-6 by LPS and OSM could also contribute to arterial lesion development. Our observations that IL-6 did not regulate HAoAF or HAoSMC in our system *in vitro* could be explained by a lack of cellular IL-6R expression by these particular cell types. As shown in other systems, the soluble form (sIL-6R), as long as in sufficient concentrations, can also enable responses of smooth muscle cells to IL-6 [28] *in vitro*. Whether sIL-6R can enable responses by HAoAF, or is present in atherosclerotic lesions at sufficient amounts, needs further study. 

VEGF, another factor implicated in the development of atherosclerosis, has been detected in atherosclerotic, but not healthy arteries, and localized to macrophages, ECs, SMCs, and within microvessels of advanced lesions [29]. In addition, atherosclerotic mice administered low doses of VEGF presented with larger lesions containing increased macrophage infiltration were compared to control mice [9]. Our findings that LPS and OSM synergized to enhance VEGF levels in the supernatants of HAoAFs and HAoSMCs suggest another mechanism by which OSM or LPS could impact inflammation and remodelling in the vascular wall by modulating local levels of VEGF.

 IL-8 is a potent neutrophil chemoattractant, and there is support for the participation of neutrophils in atherosclerosis [30]. Our findings that LPS-induced IL-8 was inhibited by OSM (Figures 1, 5, and 6) were in contrast to the effect of OSM on induction of MCP-1, IL-6, and VEGF, indicating selective regulation of genes by OSM. In other systems, OSM has been shown to inhibit IL-8 expression in response to IL-1, where human synovial and lung fibroblasts, as well as peritoneal mesothelial cells, stimulated with IL-1 showed reduced IL-8 expression upon costimulation with OSM but enhanced IL-6 and MCP-1 expression [31, 32]. The mechanism is not clear but may involve transcriptional factors that regulate the IL-8 promoter differently from promoters of MCP-1 or other chemokines. OSM does induce STAT-6 activation in lung fibroblasts [24], and STAT6 has been shown to negatively regulate the IL-8 promoter in TNF-induced keratinocytes [33]. However, whether OSM induces STAT-6 in HAoAF or HAoSMC has (to our knowledge) not yet been tested. Despite such inhibitory effects on IL-8, OSM can induce the expression of other neutrophil CXC chemokines including ENA-78, Gro *α*, and *β*, and induce neutrophil chemotaxis across EC monolayers [15]. Thus, OSM may participate in neutrophil accumulation through regulation of such other neutrophil chemoattractants. 

Analysis of activation of STAT3 showed that only OSM, and not LIF, IL-31, IL-11, nor IL-6 stimulation, resulted in elevated pSTAT3 signal, with or without added LPS, in either the adventitial fibroblast or the aortic smooth muscle cell cultures (Figure 7(a)). Furthermore, only OSM augmented HAoAF mRNA levels of MCP-1 (Figure 7(b)). Thus, the activation of STAT3 is associated with selective effects by OSM and not other gp130 cytokines in these cells. Although OSM can engage both the LIFR (gp130:LIFR-*β* complex) and the specific OSMR (gp130:OSMR-*β* complex) in human cells, the lack of comparable function by LIF in this system indicates that OSM is acting through the OSMR in both HAoAFs and HAoSMCs. The combination of OSM and LPS engaged multiple cell signalling pathways in HAoAFs (Figure 4), and coactivation of various combinations of pathways could potentiate RNA transcription or stability mechanisms in regulation of target genes MCP-1, IL-6, or VEGF. At a short term of stimulation (20 minutes), we did observe enhanced phosphorylation of p38 compared to either LPS or OSM treatment alone, and the activation of p38 has been shown to be necessary for maximal MCP-1 [34], VEGF [35], and IL-6 [36] expression in other systems. In addition, Shc, which can initiate MAPK signaling, has been shown to be activated by signaling through the OSMR*β* chain, while not being activated by signaling through the receptors of other the gp130 cytokines tested [37]. However, definitive proof as a key mechanism would need further study, for example, with pharmacological or siRNA inhibitors, as our observations associate STAT3 and augmented p38 activation with synergistic effects but do not define molecular mechanism.

 At a later time point (6 hr), OSM induced increases in CD14 mRNA (Figure 7). Since augmented CD14 has been shown to enhance LPS signalling [38], this may indicate that OSM can sensitize cells to subsequent TLR-ligand stimulation. Indeed in airway smooth muscle cells, OSM has been shown to synergize with IL-1 and IL-4 by inducing their receptor components [20, 39]. In parallel, TLR-ligand induced both the OSMR*β* and gp130 chains at the mRNA level (Figure 7), and if the mRNA elevation translates into cell surface expression, this may implicate further sensitization of HAoAF to OSM ligand present extracellularly. However, receptor change associations with the synergy require further study using molecular inhibitors to determine if they cause the altered responses.

Alternatively, the role of autocrine stimulation initiated by LPS may contribute to the mechanisms of synergy with OSM. LPS may stimulate the expression of cytokines such as IL-1 in these cells. Synergistic responses to OSM in combination with IL-1 are evident *in vitro* in a variety of cells including lung fibroblasts [31], airway smooth muscle cells [20], and chondrocytes [18].  Faffe et al. [20] showed that OSM upregulates the IL-1 receptor in airway smooth muscle cells. Further analysis would be required to determine if vascular SMC or adventitial fibroblasts respond to LPS with either IL-1 or its receptor expression, to test this possibility.

Several TLR-4 ligands have been discovered in atherosclerotic lesions including danger associated molecular patterns such as fibronectin-EDA and mm-LDL, as well as pathogen associated molecular patterns such as LPS from Gram-negative bacteria and *Chlamydia pneumoniae* HSP-60, and it has been hypothesized that TLR-4 activation could be a component of atherosclerotic lesion development [21]. In addition, activation of TLR-4 has been shown to induce OSM expression in monocytes, T cells, and dendritic cells, [40] and since OSM is detected in atherosclerotic plaques [12], such TLR activation may contribute to OSM expression locally in lesions. OSM is detected by immunolocalization in ApoE−/− mouse atherosclerotic lesions strongly in the intima earlier (20 weeks), but later (30, 54 weeks) OSM was detected more broadly in the lesion and vessel wall [12]. Thus, OSM regulation of SMC and adventitial fibroblasts may be most prominent later in the development of atherosclerotic plaque, as its levels increase over time across the vessel. Our study suggests that its presence would activate these cells to a much higher degree that other gp130 cytokines and thus contribute uniquely to pathogenic mechanisms. We suggest that exogenous or endogenous TLR-ligands, in tandem with OSM function, represent novel activities separate from other gp130 cytokines and contribute to the severity of atherosclerotic plaque development.
